# Vitamin D—The Nutritional Status of Post-Gastrectomy Gastric Cancer Patients—Systematic Review

**DOI:** 10.3390/nu14132712

**Published:** 2022-06-29

**Authors:** Tomasz Muszyński, Karina Polak, Aleksandra Frątczak, Bartosz Miziołek, Beata Bergler-Czop, Antoni Szczepanik

**Affiliations:** 1 Doctoral School of Medical and Health Sciences, Jagiellonian University Medical College, 31-530 Kraków, Poland; 2 Department of General, Oncological and Gastroenterological Surgery, Jagiellonian University Medical College, 31-501 Kraków, Poland; antoni.szczepanik@uj.edu.pl; 3 Doctoral School, Medical University of Silesia, 40-055 Katowice, Poland; m.carrine@gmail.com; 4 Chair and Department of Dermatology, Medical University of Silesia, 40-027 Katowice, Poland; ola.fratczak89@gmail.com (A.F.); bmiziolek@gmail.com (B.M.); bettina2@tlen.pl (B.B.-C.)

**Keywords:** gastric cancer, stomach cancer, vitamin D, gastrectomy

## Abstract

Gastric cancer is a malignant neoplasm of the gastrointestinal tract, with one of the standard treatment methods remaining gastrectomy. The authors conducted a systemic review of the Medline and Embase databases concerning the serum vitamin D level in post-gastrectomy gastric cancer patients, regarding all articles published until 22 May 2022 according to the PRISMA guidelines. 18 studies with a total number of 908 gastric cancer survivors were included in the analysis. The initial rate of vitamin D deficiency in gastric cancer patients undergoing gastrectomy appears to be similar to the global population deficiency. In post-gastrectomy survivors, the level of 25(OH)D may remain stable or decrease, while the level of 1, 25(OH)_2_D remains normal. Supplementation with vitamin D results in an improvement in its serum concentration and positively affects bone mineral density, which is gradually reduced in post-gastrectomy survivors. Combining vitamin D supplementation with calcium and bisphosphonates enables us to obtain better results than vitamin D and calcium only. The type of surgery influences the level of serum vitamin D and its metabolites, with total or partial gastrectomy and maintenance of the duodenal food passage remaining the most important factors. There is a strong need for randomized, controlled trials that would investigate this matter in the future.

## 1. Introduction

**Gastric cancer.** Gastric cancer is a malignant neoplasm of the gastrointestinal tract that continues to be a significant global clinical and epidemiological problem. It is estimated to be the fifth most common and third highest mortality cancer in the world, with the most dominant type of adenocarcinoma. According to gender, men are 2.2 times more likely to suffer from it. A relationship is also observed between the incidence and the region of the world with the highest prevalence and mortality in eastern and central Asia, as well as Latin America [1,2,3].

**Gastrectomy.** Gastric cancer is often diagnosed at a late stage, leading to unfavorable treatment results. In the case of early gastric cancer, 5-year survival can exceed 80%, while survival in more advanced stages decreases significantly. However, in recent decades, the incidence of gastric cancer has been declining due to the treatment of *Helicobacter pylori* infection and treatment results being better due to earlier detection and new treatment options. However, gastric resection remains a method necessary for the treatment of gastric cancer. The standard is a partial or total gastric resection with D2 lymphadenectomy (D2-LND). There are several techniques, such as endoscopic removal in the very early stages of gastric cancer, laparoscopic, robotic, and open surgery. Several gastrectomy techniques are available. Billroth I gastrectomy, called gastroduodenostomy, is a procedure in which the pylorus is removed and the distal stomach is anastomosed directly to the duodenum. In Billroth II, called a partial gastrectomy and gastrojejunostomy, a partial gastrectomy is performed and the cut end of the stomach is closed with the greater curvature of the stomach connected to the first part of the jejunum in an end-to-side anastomosis. In Roux-en-Y anastomosis (Roux-en-Y), a total gastrectomy is performed, with the small intestine connected end to end with the oesophagus, and the far end of the duodenum connected end to side with the small intestine. Systemic treatments also play an important role in the treatment of gastric cancer, including adjuvant and neoadjuvant chemotherapy, intraperitoneal chemotherapy, and chemoradiotherapy. One cannot fail to mention the latest scientific achievements: immunotherapy and targeted therapy [2,3,4,5,6,7]. 

**Nutritional status after gastrectomy.** Gastrectomy significantly affects nutritional status and exerts a change in metabolism. The main parameters of nutritional status, body weight and Body Mass Index (BMI), hemoglobin, cholesterol level, and lymphocyte count decrease significantly after surgery. The decrease in some parameters, such as body weight, glucose level, protein level, calcium level, and parameters assessing liver function remains even after a 5-year follow-up of patients undergoing gastrectomy. Research showed significant alterations in the nutritional status of post-gastrectomy gastric cancer patients. The importance of nutritional status was demonstrated in a study in which 1415 patients were examined preoperatively and postoperatively 3, 6, and 12 months after the operation. The level of albumin and the prognostic nutritional index score (PNI) decreased in the follow-up. Moreover, various parameters of the nutritional status examined perioperatively were independent prognostic factors, which illustrates the importance of research into the nutritional status of patients after gastrectomy due to cancer. Similar conclusions were drawn in a study published in Annals of Surgical Oncology in 2018, malnutrition 12 months after gastrectomy significantly and negatively affected the overall survival of patients with gastric cancer. A study focused on the long-term 5-year follow-up of hematological parameters and nutritional status in patients after gastric resection for cancer, published in 2017 in the Journal of Gastrointestinal Surgery, showed that ferritin and triglycerides levels gradually decreased after gastrectomy, while other parameters of nutritional status declined slightly or remained at a constant level [8,9,10,11].

**Vitamin D.** One of the indicators that should be considered in the context of nutrition for gastric cancer patients is the serum vitamin D level. Vitamin D is a fat-soluble vitamin obtainable from the diet, either in the form of vitamin D_2_ (ergocalciferol) or vitamin D_3_ (cholecalciferol). Vitamin D_3_ is also synthesized from 7-dehydrocholesterol by ultraviolet B (UVB, 290–320 nm) radiation in the epidermis of the skin. Both D_2_ obtained from the diet and D_3_ from the skin or gastrointestinal tract bind in circulation to the vitamin D binding protein (VDBP) and are delivered to the liver, which is the first place in a two-step metabolism, where vitamin D 25-hydroxylase transforms the delivered metabolites into 25(OH)D (calcidiol). Calcidiol is the dominant vitamin D form in the serum. However, it is further metabolized by 25(OH)D 1α-hydroxylase in the proximal tubules of the kidneys into 1α,25-dihydroxyvitamin D (1α,25(OH)_2_D)—calcitriol, which is the most biologically active form of vitamin D that binds to the vitamin D receptor (VDR) in tissues, allowing it to perform its biological functions. 25(OH)D can also be transformed by 25-hydroxyvitamin D_3_-24-hydroxylase into 24,25-dihydroxyvitamin D_3_—24,25(OH)_2_D_3_, which is considered a metabolite of 25(OH)D showing weak activity. The level of vitamin D deficiency is ≤50 nmol/L 25(OH)D according to the guidelines of the Endocrine Society Task Force on Vitamin D [12,13].

Although many articles discuss vitamin D level in the context of risk of gastric cancer development or changes in bone density after gastrectomy leading to osteomalacia or osteoporosis, hardly any investigate changes in serum vitamin D and its concentration of metabolites in gastric cancer survivors after gastrectomy. The purpose of our paper was to provide a systematic review of the available studies on vitamin D levels in patients post-gastrectomy due to gastric cancer. 

## 2. Materials and Methods

**Figure 1:**
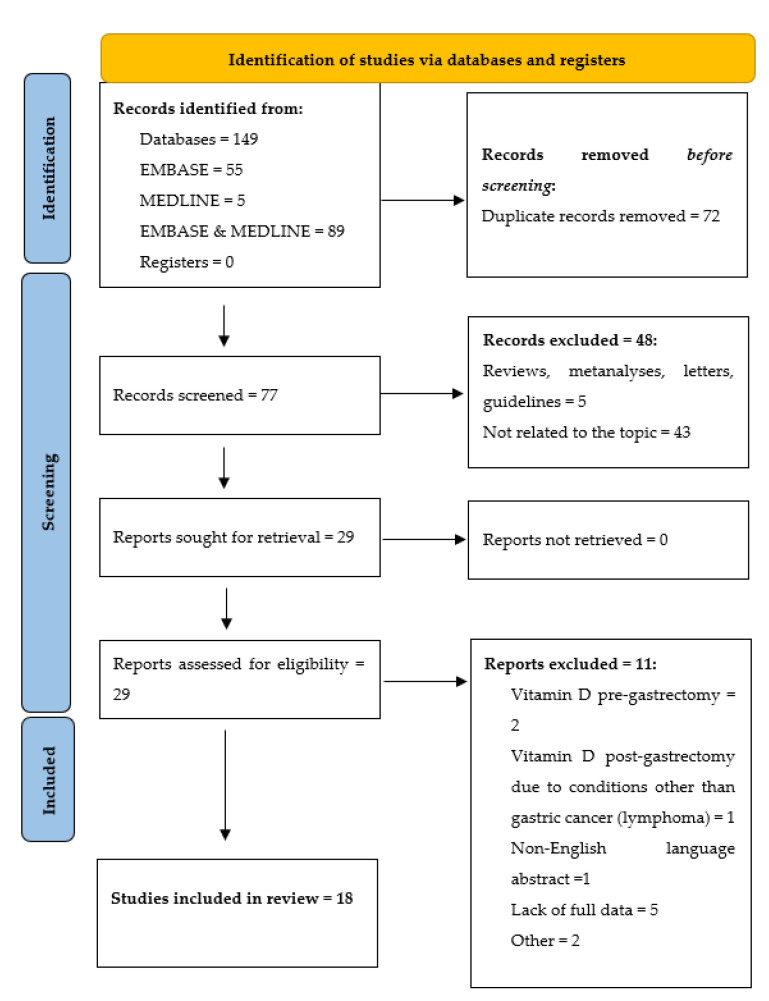
Identification of studies via databases and registers according to the PRISMA guidelines.

The systematic review was carried out according to the PRISMA guidelines (Figure 1) The authors conducted research in Medline and Embase databases, using MeSH and Emtree terms: “stomach cancer”, “gastric cancer”, “gastrectomy”, “vitamin D”, “25-hydroxyvitamin d”, “cholecalciferol derivative”, “cholecalciferol”, “calcitriol “, “24,25 dihydroxyvitamin”; including all types of articles published until 22 May 2022, including data on the type of study, population, intervention, and outcome. 55 records in Embase, 5 in MEDLINE, and 89 records in both databases were found. After removing duplicates, the articles were manually searched to fulfill the inclusion criteria. Only original studies, case reports, or case series that had at least an abstract published in English, on human subjects who underwent gastrectomy due to gastric cancer and had their serum vitamin D levels determined at least once after gastrectomy were included in the review. 

## 3. Results

Eighteen original studies or case reports on serum vitamin D status were identified in post-gastrectomy patients due to gastric cancer, with a total number of 908 gastric cancer survivors included (Table 1).

The majority of the patients were men. The first report was made by Kobayashi et al. in 1994 [31], while the newest data were reported by Atsumi et al. in 2021 [14]. The investigated vitamin D metabolites were 25(OH)D (11 studies) [14,17,18,19,21,23,24,25,26,28,31], 1,25(OH)_2_D (6 studies) [14,17,24,25,27,31], and 24,25(OH)_2_D (2 studies) [24,31]. Six studies did not report the investigated metabolite [15,16,20,22,29,30], declaring that they analyzed “serum vitamin D”. The other concomitant and most frequently analyzed parameters were bone mineral density (nine studies) [14,16,17,19,20,21,23,26,30], serum estradiol level (three studies) [14,17,25] and other bone metabolism biomarkers, as part of the studies focused on bone density and risk of osteoporosis after gastric cancer surgery with vitamin D and its metabolites evaluated only as indicators. Five studies reported the type of surgery with the declared techniques Billroth I, Billroth II, and Roux-en-Y [18,24,25,28,31]. Seven out of 18 studies analyzed changes or serum vitamin D concentration 12 months after surgery [14,16,17,18,19,22,23]; the longest reported time from surgery to analysis was 18 years [29]. 

In seven out of 18 studies, vitamin D had been supplemented in patients with gastric cancer who underwent gastrectomy [15,16,19,21,22,26,27]. However, only two studies reported the doses administered, ranging from 1000 IU of cholecalciferol per day with calcium or 70 mg/week of alendronate [16] to 16,000 IU of vitamin D_3_ administered every 10 days [19]. Virik [21], Ribeiro [22], Cuerda [26], Veeralakshmanan [15], and Rino [27] only reported that patients were supplemented with vitamin D and/or calcium, multivitamin and mineral supplement, or 1α(OH)D_3_, with no report of daily doses, the regimen of administration, type of supplementation, or information whether it was advised by the physician or performed by the patient himself. The reported supplementing time ranged from 12 to 18 months [15,16,19,21,22,26,27].

Several studies compared vitamin D concentration both at baseline and at specific time points after surgery. The initial deficiency was present in 36.67% of the participants in the study by Veeralakshmanan et al. [15]; Virik et al. reported that at baseline, 24/27 (89%) patients had vitamin D levels < 50 nmol/L (mean 35.3 nmol/L) and 26/27 (96%) had vitamin D levels < 75 nmol/L (mean 38.4 nmol/L) with a normal range from 50 nmol/L to 125 nmol/L [21]. In contrast, Rino Y. et al. found that the level of 1,25(OH)_2_D was normal in all patients; however, 25(OH)D was below the normal range in 7 of the 22 patients (31.8%) [24]. The findings on serum vitamin D concentration levels after gastrectomy are ambiguous. Atsumi et al., who performed research on the level of 25(OH)D after gastric resection due to cancer, reported in 2019 a significant decrease in serum concentration 24 months after surgery [17]. His findings were confirmed by Jeong et al., whose study found that the vitamin D level was lower in gastric cancer survivors (20.3 ± 0.5 IU vs. 17.5 ± 1.2 IU) than in healthy individuals [20]. Ribeiro reported that in his study on fat-soluble vitamins, vitamin D was the only deficient one in 82% of patients, and the deficiency remained high throughout the entire period of follow-up, which lasted up to 24 months after surgery [22]. Rino et al. investigated the topic in two studies: they reported that the crucial time for the change in serum vitamin D concentration is 12 months, as the mean serum level of 25(OH)D was significantly lower in patients at 1 year or more postoperatively than in those at less than 1 year postoperatively. The prolonged analysis, performed on the group of patients who underwent gastrectomy due to gastric cancer less than 10 years before the study, showed that 25(OH)D was reduced in 6 of 21 patients (29%) [24,25]. In the study by Cuerda et al., 25(OH)D deficiency was initially observed in 45% of the patients. However, 25(OH)D concentration depends on the type of surgery, with a higher level in patients belonging to the pouch group than Roux-en-Y reconstruction (47.3 nmol/L compared to 33.9 nmol/L, respectively). Another study concerning the level of 24,25(OH)_2_D, a weak activity metabolite of 25(OH)D, showed that after a year from gastrectomy, its level was below the normal range in 19 of the 22 patients (86%), and analyzing survivors with gastrectomy who underwent surgery less than 10 years prior to the study, 24,25(OH)_2_D was reduced in 17 patients (81%) [26]. Kobayashi also reported a decrease in serum 24,25(OH)_2_D concentration, as well as its dependence on the type of surgery, with patients who underwent Billroth II manifesting lower 24,25(OH)_2_D levels than those with Billroth I [31].

However, the findings of several authors suggest that gastric cancer gastrectomy does not affect serum vitamin D concentration levels, or at least some of its metabolites, as stated previously. A study by Atsumi et al. in 2021 showed that the concentration levels of 25(OH)D, 1,25(OH)_2_D remained stable 12 months after the surgery [14]. In 2019, the same author reported that despite surgery, 1,25(OH)_2_D levels were consistently in the normal range up to two years of follow-up [17]. The findings of Baek seem to confirm this trend, as the investigated level of 25(OH)D did not change significantly compared to the baseline at 12 months after surgery [23]. Two studies by Rino in 2007 showed that the level of 1,25(OH)_2_D was normal in all patients, as well as in those who had undergone gastrectomy for gastric cancer and had been followed for even 10 years [24,25]. Schmidel et al., who analyzed the serum vitamin D concentration level in patients 3–18 years after gastrectomy, stated in their study its serum level remained normal [29].

None of the studies reported an increase in serum vitamin D levels in all patients; the reported improvements were relative to another study group or related to supplementation. In patients undergoing Billroth II surgery, 24,25(OH)_2_D concentrations were reported to be reduced, while 25(OH)D and 1,25(OH)_2_D concentrations increased [31]. Toyomasu reported that serum vitamin D in the Billroth-I group was significantly higher than in Roux-en-Y patients [18]. This remains consistent with the findings of Iivonen, who observed that patients with Roux-en-Y tended to have lower serum vitamin D concentrations than patients in the pouch group [28]. The passage of food through the duodenum also affected the results obtained. Rino Y. found, that the 1,25(OH)_2_D/25(OH)D ratio was significantly higher in the patients without passage of food through the duodenum due to the reconstructive method, while the 25(OH)D/24,25(OH)_2_D ratio was significantly higher in the patients with remaining duodenal food passages [25]. In another study, he proved that the serum level of 25(OH)D was significantly lower in patients who had received total gastrectomy than in patients who underwent other gastrectomy procedures [24].

The issue of vitamin D supplementation after gastrectomy has not been fully elucidated in all studies; however, in every reported case, a positive influence on serum vitamin D level was demonstrated. In a study by Veeralakshmanan, supplementation for 18 months with multivitamin tablets did not only significantly improve vitamin D levels, but also ferritin, folate, and vitamin B_12_ deficiencies [15]. A study by Climent M. showed, that even shorter periods of supplementation may result in positive outcomes, as providing the patients with 16,000 IU of vitamin D_3_ every 10 days over 3 months made 35 out of 40 patients included in the study reach values of 25(OH)D over 30 ng/mL, while over 12 months of vitamin D supplementation caused 38 patients to achieve serum vitamin D levels within the normal range of 25(OH)D [19]. Supplementation with vitamin D and calcium provided by Virik in his study resulted in an evaluation of the mean serum vitamin D level of 81.1 nmol/L (range 34–147). Throughout the study period, vitamin D levels improved to >50 nmol/L in 26/27 (96%) patients and to >75 nmol/L in 13/27 (48% of patients) [21]. In a study by Ha et al., 1000 IU of cholecalciferol had been administered per day with calcium or 70 mg/week of alendronate. The results of the study, investigating the prevention of bone loss in post-gastrectomy gastric cancer survivors, showed that supplementation with both vitamin D and calcium was less effective than treatment of vitamin D and calcium combined with bisphosphonate [16]. However, the study by Rino in 2000 showed that the severity of metabolic bone disorder analyzed by the MD/MS method improved after only 1α(OH)D treatment in 56.3% of patients [27]. Wu in his case report also stated that in a patient with osteomalacia secondary to vitamin D deficiency after gastric cancer gastrectomy, supplementing the patient with vitamin D caused the appearance of repeated bone scintigram to normalize [30]. The question of the change in bone density after gastrectomy appears to bring important information on the change in bone metabolism [32], as in the study by Atsumi in 2019, where BMD decreased significantly by 0.04 ± 0.03 g/cm^2^ 12 months after gastrectomy and by 0.05 ± 0.04 g/cm^2^ 24 months after gastrectomy [17]. The same author in 2021 reported that BMD decreased by median degrees of 3.4% and 3.9% in male and female patients 12 months after surgery [14]. Jeong proved in his study that gastric cancer survivors who underwent gastrectomy have a significantly higher risk of osteopenia (RR = 2.90) and osteoporosis (adjusted RR = 4.63) than the healthy population [20].

## 4. Discussion

**Gastric cancer and vitamin D deficiency as a risk factor for its development.** Gastric cancer is a malignant neoplasm of the gastrointestinal tract which, despite the constant efforts of doctors, scientists, and the health care system, continues to be a significant clinical and epidemiological problem in the world [1]. The risk factors for gastric cancer are: *Helicobacter pylori* infection; Ebstein–Barr viral infection; gastric ulcer disease; smoking and alcohol consumption; exposure to dust; high temperature; metals such as chromium; a diet rich in salt and N-nitroso compounds; obesity; pernicious anemia; blood group A; and even genetic syndromes such as familial adenomatous polyposis (FAP); Lynch syndrome; interleukin-17 (IL-17) and interleukin-10 (IL-10) polymorphisms of interleukin genes; hereditary diffuse gastric cancer (HDGC) and gastric adenocarcinoma; and proximal polyposis of the stomach (GAPPS) [2,3]. Epidemiologic evidence supports the role of vitamin D and vitamin D receptor (VDR) polymorphisms in the risk of several cancers, 1,25(OH)_2_D affecting cell differentiation and growth, as well as the appearance of invasion, angiogenesis, and metastasis in certain types of cancer [33]. At the same time, gastric cancer is a cancer that is extremely dependent on lifestyle; therefore, research on the potential role of nutrients, including vitamin D, was carried out, to investigate the matter of their influence on the prevalence of this malignancy. A systematic review published by Khayatzadeh et al. in 2015 on the relationship between vitamin D intake, serum vitamin D level, and risk of gastric cancer, found no evidence of a significant association between vitamin D status and risk of gastric cancer [34]. However, further studies by Tagliabue et al., which focused on the role of vitamin D and the VDR, found a borderline decrease in cancer risk for subjects with high levels of vitamin D binding protein compared those with low levels [33]. The latest reports show that vitamin D may be related to an anticancer mechanism of action in gastric cancer development by regulating epigenetic pathways, affecting the expression of microRNAs (miRNAs), accelerating the effect of cisplatin, and regulating intracellular signal transduction. Shah et al., who investigated this matter, found that serum vitamin D level is lower in patients suffering from *Helicobacter pylori* infection than those unaffected. Furthermore, patients suffering from vitamin D deficiency were found to have fewer chances to eliminate bacteria than those with normal vitamin D levels. UVB radiation, which promotes vitamin D synthesis in the skin, was found to reduce the incidence and mortality rate in patients with gastric cancer with an adequate serum vitamin D level related to a better survival rate. Also, the polymorphisms in the vitamin D receptor gene were found to be related to an increased risk of gastric cancer [35]. Vitamin D may modulate the expression of some miRNAs that are deregulated in gastric cancer cells. In this mechanism, vitamin D reduces chemoresistance at the cellular level [36]. Our review found that patients with gastric cancer have vitamin D deficiency, with reported rates ranging from the initial 36.67% of patients [15] up to 89% [21]. In patients with gastric cancer, it was also the vitamin D metabolite investigated that mattered, as demonstrated by Rino Y et al. al, who found that although 1,25(OH)_2_D may be normal in gastric patients, the level of the most active metabolite, 25(OH)D, may remain below the normal range in a significant number of them (31.8%) [24]. However, there is still no single study that would support with strong evidence that vitamin D supplementation could result in a decrease in gastric cancer incidence rate neither in the general population nor in relapsed patients [36], and the deficiency reported initially in gastric cancer patients remains consistent with the general global population deficiency, estimated at 37.3% (circulating concentrations of 25(OH)D below 20 ng/mL) [37].

**Vitamin D level in post-gastrectomy gastric cancer survivors.** The results of the provided studies remain ambiguous. The observational studies that reported serum vitamin D levels or its metabolites compared to baseline, without supplementation or relative concentration versus different types of surgery, etc., seem to indicate that in post-gastrectomy gastric cancer survivors the level of 25(OH)D may remain normal or decrease [14,17,18,19,21,23,24,25,26,28,31], while the level of 1,25(OH)_2_D remains normal [14,17,24,25,27,31].

**Vitamin D post-gastrectomy supplementation.** Most available research on the influence of gastrectomy on serum vitamin D levels is focused on patients with bariatric surgery. As reported by Jamil et al., patients with obesity may suffer from vitamin D deficiency due to vitamin D sequestration in fat, decreased sun exposure, sedentary lifestyle, and the psychological component of covering more skin with vitamin D deficiency, the most common observed vitamin deficiency in patients prior to surgery treating obesity. Most studies indicate, however, that in obese patients undergoing bariatric surgery, vitamin D deficiencies do not develop de novo postoperatively, but rather are connected to the preoperative status and improve with supplementation [38]. Unfortunately, current Clinical Practice Guidelines on vitamin D supplementation in bariatric surgery differ between societies, with most recommending high doses of vitamin D supplementation following surgery, ranging from 3000 IU daily to 50,000 IU 1–3 times weekly, and increasing to 50,000 IU 1–3 times daily in case of severe malabsorption. However, it should be noted that they do not fulfill the criteria for optimal guideline development, in part possibly due to limited resources, and are based on expert opinions [39]. In our review, concerning post-gastrectomy gastric cancer patients, vitamin D supplementation has been implemented in seven of 18 studies [15,16,19,21,22,26,27], three of which reported the initial deficiency [15,21,24]. In all studies, vitamin D supplementation positively affected its serum level or bone mineral density; however, no strict recommendations on dose or regimen were made. Only a study by Ha et al. proved that in post-gastrectomy gastric cancer survivors with bone loss, it is more effective to combine vitamin D supplementation with calcium and bisphosphonates than calcium alone [16]. However, it should also be noted, that supplementing patients who suffer from a lack of calcium and vitamin D, resulting for example in osteoporosis, may also bring some dangers. Chiodini and Bollard provided a review of the risk-benefit profile of such supplementation with calcium, based on large randomized controlled trials, with a conclusion, that its benefit in preventing fractures (with or without vitamin D) is at most very small, if any. Also, the possible adverse events outweigh the possible benefits of such supplementation [40]. A study by Park et al. on a Korean population investigated the association between calcium supplementation and cardiovascular outcomes and showed that the cumulative incidence of acute myocardial infarction, ischemic stroke, and death was significantly higher in the calcium supplementation group than in the control group [41]. These findings were confirmed by the Women’s Health Initiative Calcium/Vitamin D Supplementation Study, which showed that calcium supplements with or without vitamin D modestly increased the risk of cardiovascular events, especially myocardial infarction in women [42]. The possible side effects of calcium supplementation may also include possible mild, but common gastrointestinal side effects [40], which may be especially important in the group of post-gastrectomy cancer patients.

As the composition of dietary supplements varied across the presented interventional studies, it should also be taken into consideration that it is not only calcium that is related to vitamin D metabolism. Vitamin K_2_ and magnesium both appear to be involved in bone metabolism with data suggesting that vitamin K_2_ supplementation might improve bone quality and reduce fracture risk in osteoporotic patients [43]. In the meta-analysis of eight randomized controlled trials including a total of 971 subjects, vitamin K combined with vitamin D significantly increased the total bone mineral density, with the most favorable effect expected for vitamin type K_2_ [44]. Continuous combination therapy with vitamin K_2_ and D_3_ was proved to be useful for increasing vertebral bone mass in postmenopausal women, demonstrating superiority over vitamin K_2_ alone [45]. Dai Qi et al. found in their original study, concerning the influence of magnesium supplementation on the vitamin D metabolism, that optimal magnesium status may be important for optimizing 25(OH)D status, as it increased the 25(OH)D concentration when baseline 25(OH)D concentrations were close to 30 ng/mL, but decreased it when baseline 25(OH)D was higher (from ∼30 to 50 ng/mL) [46]. All of this information outlines the complexity of vitamin D metabolism and indicates a strong need for further studies that could investigate whether post-gastrectomy gastric cancer patients should receive vitamin supplementation and what composition it should have, also in the context of diet. As it is known that food intake accounts for a smaller contribution to 25(OH)D concentration than does solar UVB exposure and supplements, it should be noted that meat is a good source of vitamin D as 25(OH)D. The EPIC-Oxford study, which investigated plasma 25(OH)D concentrations in meat eaters, fish eaters, vegetarians, and vegans found them lower in vegetarians and vegans than in meat and fish eaters [47]. A study by Liu et al. assessed the possible dietary daily intake of vitamin D in an Australian population, providing data that meats, chicken, fish, eggs, and dairy produce, may alone have contributed about 4.2 μg vitamin D equivalents per day to average Australian diets of adults > 18 years in 1995 and 4.3 μg in 2011–2013 [48]. The influence of undergoing gastrectomy, with its possible results concerning the recovery period and further impact on the type of used diet, should also be taken into consideration in further studies.

**Bone mineral density loss in post-gastrectomy gastric cancer survivors.** Oh et al. investigated the matter of bone mineral density and osteoporosis risk in post-gastrectomy patients in their meta-analysis, including 1204 patients. Factors leading to decreased bone mineral density in this population were calcium malabsorption, secondary hyperparathyroidism, and dominant bone resorption with a pooled incidence of decreased BMD estimated at 36%. It was also found that calcium and 25(OH)vitamin D levels were significantly decreased, while PTH and 1,25(OH)D levels increased significantly in the gastrectomy group compared to the control group [49]. Their findings remain consistent with those obtained in this review: in all studies investigating BMD after gastrectomy in gastric cancer survivors, it was decreased [14,16,17,19,20,21,23,26,30]. Vitamin D supplementation improved the observed BMD in studies including this intervention [15,16,19,21,22,26,27]. Bone mineral density loss may be only one of many changes related to vitamin D and calcium metabolism. In 2019, Choi et al. published a study based on a Korean population, which showed that gastric cancer patients who received a total gastrectomy had an increased incidence of Alzheimer’s disease, with vitamin B_12_ deficiency probably playing a crucial role [50]. However, there are significant associations between vitamin D deficiency and both dementia and Alzheimer’s disease [51,52]. The authors outlined a need for further studies in order to investigate if vitamin D deficiency may influence Alzheimer’s disease incidence rate in post-gastrectomy gastric patients, along with vitamin B_12_.

**Type of surgery and serum vitamin D level.** Although the techniques implemented in bariatric surgery and gastrectomy due to gastric cancer may differ, with sleeve gastrectomy performed in bariatric surgery as one of the possible options, some of them remain common, e.g., Roux-en-Y technique. A systematic review and meta-analysis performed by Salman et al., including 717 patients who underwent one of these procedures with 50.63% of the patients in the Roux-en-Y arm, investigating the influence of gastric bypass and sleeve gastrectomy on bone mineral density and bone turnover markers showed that there was no significant difference in bone mineral density between these types of surgery. Only bone alcaic phosphatase and parathormone levels were significantly higher after Roux-en-Y surgery [53]. Howevr, this interesting finding, pertaining to the influence of the type of surgery on postoperative serum vitamin D levels, does not fit our findings. In our review, in gastric cancer survivors, the type of gastrectomy affected the results obtained. By comparing the techniques reported, Billroth II resulted in statistically significant higher levels of 1,25(OH)2D and reduced levels of 24,25(OH)2D versus the Billroth I group [31]. However, the Billroth I technique resulted in better vitamin D serum levels compared to the Roux-en-Y technique up to 2 years after surgery [18], with Roux-en-Y technique also remaining inferior to the pouch group in maintaining serum vitamin D concentrations [28]. It is the passage of food through the duodenum that appears to influence the serum vitamin D level, as the ratio 1,25(OH)_2_D/25(OH)D remains significantly higher in patients without the passage of food through the duodenum [25]. Total gastrectomy results in a lower serum vitamin D level than other gastrectomy procedures [24]. The relationship between vitamin D level and surgery has been raised for many years, for example in the context of the association between perioperative vitamin D status and outcomes after surgery, as it is known that some factors affecting the operation outcome, e.g., the inflammatory state (assessed for example by CRP level), is differentially modulated by co-existing infections and vitamin deficiencies [54]. A systematic review by Iglar and Hogan provided that vitamin D hypovitaminosis is associated with adverse outcomes after diverse surgical procedures [55]. Barker et al. conducted a study, in which patients undergoing open-heart surgery were randomly assigned to one of two groups, receiving either cholecalciferol (50,000 IU/dose) or placebo on three separate occasions: orally the evening before surgery and either orally or per nasogastric tube on postoperative days 1 and 2. Supplemental vitamin D prevented the sudden decrease in 25(OH)D induced by open-heart surgery during postoperative care. What is more, plasma 25(OH)D gradually increased from baseline to day three and remained significantly increased thereafter but plateaued to discharge with supplemental vitamin D [56].

**Limitations.** The authors outline the heterogeneity of the studies included in the review, ranging from case reports to interventional studies with different groups, abundance, and types of patients. It was not for all of the studies that satisfactory data on the type, intervention, comparison, and outcome of the patients were provided. Laboratory results were not always provided with particular serum vitamin D metabolites. Also, data on the type and dosage of vitamin D supplementation implemented in interventional studies are lacking. The research also did not report exact pathological findings on the type of gastric cancer, its staging, and grading.

## 5. Conclusions

The initial rate of vitamin D deficiency in gastric cancer patients undergoing gastrectomy seems to be similar to the global population deficiency. In post-gastrectomy survivors, the level of 25(OH)D may remain stable or decrease, while the level of 1,25(OH)_2_D remains normal. Supplementation with vitamin D results in an improvement in its serum concentration and positively affects bone mineral density, which is gradually reduced in post-gastrectomy survivors. Combining vitamin D supplementation with calcium and bisphosphonates enables improving bone mineral density results; however, no specific strong recommendations have been made regarding dosage and supplementation regimen. The type of surgery influences the level of serum vitamin D and its metabolites, with total or partial gastrectomy and maintenance of the duodenal food passage remaining the most important factors. There is a strong need for more randomized, controlled trials that would investigate this matter in the future.

## Figures and Tables

**Table 1:** Original studies and case reports on serum vitamin D concentration in patients with gastric cancer after gastrectomy.

No of Study	Year	Author	Population	Study	Results (Vitamin D, Additional Findings)
1.	2021	Atsumi Y. et al. [14]	47 patients (38 men and 9 women) with early gastric cancer	bone mineral density (BMD), serum levels of 1,25-dihydroxy vitamin D (1,25(OH)2D), 25-hydroxy vitamin D (25(OH)D), and estradiol (E2) were measured before and after surgery to investigate gender-related differences in bone metabolism changes after gastric cancer surgery.	12 months after surgery: serum 25(OH)D and 1,25(OH)_2_D levels remained nearly within the normal range throughout the observation period in both male and female patients. BMD decreased by 3% in male and female patients, and the serum level of E2 in male patients increased by 22 pg/mL.
2.	2020	Veeralakshmanan P. et al. [15]	63 patients after esophagectomy and/or subtotal/total gastrectomy for malignant tumors	patients were screened for vitamin D, ferritin, folate, and vitamin B12 deficiency; median follow-up after 18 months, while all patients included in the study received multivitamin tablets from their first visit.	37% of the patients were deficient in vitamin D at the screening. After follow-up: vitamin D levels improved significantly due to supplementation. Ferritin, folate, and vitamin B12 deficiencies at baseline—43%, 10%, 6%—levels improved significantly after supplementation.
3.	2020	Ha J et al. [16]	107 patients with gastric cancer	Participants randomly assigned to receive 70 mg of alendronate at a weekly dose and daily elemental calcium (500 mg) with cholecalciferol (1000 IU) or daily elemental calcium (500 mg) with cholecalciferol (1000 IU) only for 12 months starting one week after surgery	At baseline, there were no differences between the two groups in bone mineral density. A significant decrease was observed in the control group at the end of the study—supplementation with vitamin D and calcium only was less effective in preventing bone loss than treatment combined with bisphosphonate.
4.	2019	Atsumi Y et al. [17]	39 men with early gastric cancer	Dual-energy X-ray absorptiometry (DEXA) was performed to measure BMD of the lumbar spine before and 12 and 24 months after surgery; Alkaline phosphatase (ALP), 1,25(OH)_2_D, 25(OH)D, and E2 were measured before surgery and every 3 months until 24 months after surgery.	The serum 25 (OH) D level decreased significantly by 5 ± 6 ng/mL 24 months after surgery, and the serum 1,25(OH)_2_D levels were consistently in the normal range. BMD decreased significantly by 0.04 ± 0.03 g/cm^2^ 12 months after gastrectomy and by 0.05 ± 0.04 g/cm^2^ 24 months after gastrectomy; ALP increased significantly by 38 ± 104 IU/L 24 months after surgery; serum level of E2 increased significantly by 33 ± 7 pg/mL 12 months after gastrectomy. Lower preoperative BMI was significantly correlated with the reduction in BMD 12 months after surgery.
5.	2018	Toyomasu Y. et al. [18]	156 patients who underwent laparoscopic distal gastrectomy due to gastric cancer with Billroth-I or Roux-en-Y reconstruction	The authors compared hemoglobin, ferritin, serum iron, vitamin B12, 25(OH)D, body weight, and gastric emptying after laparoscopic distal gastrectomy in patients with Billroth-I or Roux-en-Y reconstruction.	Serum vitamin D in the Billroth-I group was significantly higher at 1 year and 6 months, 1 year and 9 months, and 2 years after surgery. >6 months after surgery: hemoglobin levels in the Billroth-I group were significantly higher. >9 months after surgery: ferritin levels were significantly higher in the Billroth-I group. Gastric emptying was significantly slower after Roux-en-Y reconstruction.
6.	2017	Climent M. et al. [19]	40 disease-free patients at least 24 months after gastric cancer resection	Serum markers of bone metabolism (C-terminal cross-linked telopeptide of type I collagen, serum concentrations of bone-specific alkaline phosphatase and osteocalcin) were evaluated at baseline and at 3 and 12 months, BMD and the presence of fractures were evaluated by radiograph at baseline; patients with 25(OH)D ≤30 ng/ml at baseline received 16,000 IU of vitamin D_3_ every 10 days during the 1-year follow-up.	>3 months of vitamin D supplementation: 35 patients reached 25(OH)D values greater than 30 ng/ml. >12 months of vitamin D supplementation: 38 patients were in the normal range of 25(OH)D. >12 months: levels and markers of bone turnover decreased significantly after vitamin D intervention
7.	2019	Jeong S.M. et al. [20]	Gastric cancer survivors (103) who had a history of gastric cancer in the questionnaire	8156 people over 50 years of age who have been tested with DEXA served as the control group, 103 gastric cancer survivors who had a history of gastric cancer in the questionnaire as the study group; After adjusting for sex, age, BMI, smoking status, alcohol consumption, physical activity, and bone health-related history (history of fracture or family history of osteoporosis), risk of osteopenia, osteoporosis was assessed and compared.	Vitamin D level was lower in gastric cancer survivors (20 ± 11 IU vs. 18 ± 1 IU). After adjusting for the assessed criteria, there was a significantly higher risk of osteopenia (relative risk, RR = 2.90) and osteoporosis (adjusted RR = 4.63) in gastric cancer survivors.
8.	2016	Virik K. et al. [21]	30 post-gastrectomy patients	Single-centered study in Australia: patients who underwent total (12) and partial/distal gastrectomy (12), with six having subtotal gastrectomy (22 [73%] had Roux-en-Y or Billroth-II reconstruction and eight had a Billroth-I/other type of reconstruction) were evaluated for age, gender, pathology, type of surgery, 25(OH)D, calcium, parathyroid hormone, BMD, vertebral XR, urinary calcium, and N-telopeptides of type I collagen, corticosteroid use, alcohol intake, hyperthyroidism, menopausal status, hyperparathyroidism, and preexisting bone disease. Supplementation with vitamin D and calcium has been introduced and the parameters have been re-assessed after supplementation.	At baseline, 24/27 (89%) patients had vitamin D levels <50 nmol/L (mean 35) and 26/27 (96%) had vitamin D levels <75 nmol/L (mean 38). Mean levels after vitamin D and calcium supplementation: vitamin D = 81 nmol/L (range 34–147), vitamin D levels improved to >50 nmol/L in 26/27 (96%) patients and to >75 nmol/L in 13/27 (48%). Poor bone health and vitamin D deficiency were clinically significant problems post-gastrectomy, vitamin D deficiency and a secondary elevated level of parathormone were common.
9.	2014	Ribeiro U. et al. [22]	82 subjects (71 post-esophagectomy and 11 after total gastrectomy)	All patients attending the surgical outpatient clinic who had undergone either an esophagectomy or total gastrectomy ≥12 months ago had a blood sample taken for laboratory tests and the number of months after surgery, nutritional supplementation, and their use of pancreatic enzyme replacement therapy (PERT) recorded. 37% of the patients were taking regular multivitamin and mineral supplements and 9% PERT.	Vitamin D was the only fat-soluble vitamin deficient (82%) and the deficiency remained high throughout the follow-up period. Deficiency was identified for iron (17%), serum ferritin (27%), and iron saturation (38%). Mineral deficiencies were found in zinc (51%), selenium (39%), and calcium (10%). The percentage of patients deficient in iron, ferritin, and iron saturation rates increased with time, while deficiencies in selenium and calcium were highest 12–24 months after surgery, then decreased slightly.
10.	2008	Baek K.H. et al. [23]	36 patients (24 men, 12 women)	36 patients (58 ± 11 years) who had DEXA performed before and 12 months after gastrectomy. Blood was sampled from all patients to determine serum calcium, phosphorous, and bone turnover markers levels before gastrectomy and at 1, 3, 6, and 12 months after surgery and for serum parathyroid hormone (PTH) and 25(OH)D levels before and 12 months after surgery.	The 25(OH)D level at 12 months after surgery did not change significantly compared to baseline. PTH levels increased by a mean of 64% at 12 months compared to baseline. Significant correlations were found between the percentage change in bone mass density in the lumbar spine and total hip and the percentage change of PTH level from baseline to 12 months.
11.	2007	Rino Y. et al. [24]	22 patients (17 men, 5 women)	Laboratory tests were performed to examine the following parameters: 1,25(OH)_2_D; 25(OH)D; 24,25(OH)_2_D; ionized calcium; calcium; phosphorus; alkaline phosphatase; N-parathyroid hormone; and osteocalcin.	The level of 1,25(OH)_2_D was found to be normal in all patients. The 25(OH)D level was below the normal range in seven of the 22 patients (32%). The mean serum level of 25(OH)D was significantly lower in patients at 1 year or more postoperatively than the level in those at less than 1 year postoperatively and significantly lower in patients who had received total gastrectomy than in patients who underwent other gastrectomy procedures. The level of 24,25(OH)_2_D, a 25 (OH)D metabolite showing weak activity, was below the normal range in 19 of the 22 patients (86%).
12.	2007	Rino Y. et al. [25]	21 patients who had undergone gastric cancer gastrectomy and have been followed for less than 10 years	The bone disorders of the patients were assessed by micro- densitometry. The levels of 1,25(OH)_2_D, 25(OH)D, 24,25(OH)_2_D, N-terminal parathormone, calcitonin, E2, osteocalcin, and ALP were measured	The level of 1,25(OH)_2_D was normal in all patients, while 25 (OH) D was reduced in six out of 21 patients (29%); 24,25(OH)_2_D was reduced in 17 patients (81%). The 1,25(OH)_2_D was significantly higher in the patients with grade I to III bone disorder compared to patients with normal bones or early bone disease. The 1,25(OH)_2_D/25(OH)D ratio was significantly higher in patients without passage of food through the duodenum due to the reconstructive method, while the 25(OH)D/24,25(OH)_2_D ratio was significantly higher in the patients with remaining duodenal food passage. Bone disorder was found in nine out of 21 patients (43%).
13.	2007	Cuerda C. et al. [26]	54 patients (27 men and 27 women)	The patients were retrospectively followed for more than 12 months after surgery with a nutritional evaluation that included anthropometry, biochemical data, and bone mineral density measurement by dual-energy X-ray absorptiometry monitored.	The incidence of 25(OH)D deficiency and secondary hyperparathyroidism was 45% and 76%, respectively. Patients had been supplemented with vitamin D (17%), iron (43%), B_12_ (87%), and calcium (18%).
14.	2000	Rino Y. et al. [27]	16 patients	The purpose of this study was to elucidate the efficacy of 1α(OH)D supplementation for the treatment of metabolic bone disorder after gastrectomy.	The severity of the metabolic bone disorder analyzed using the modified micro densitometry (MD/MS) method improved after 1α(OH)D supplementation treatment in 56% of patients.
15.	2000	Iivonen M.K. et al. [28]	51 patients	Patients were operated on and randomized after total gastrectomy and one of two types of reconstruction. Twenty patients with jejunal pouch reconstruction and 14 patients with Roux-en-Y reconstruction (67% of all) survived at least 3 years after total gastrectomy. Symptoms, eating capacity, blood haemoglobin, serum albumin, and transferrin were evaluated during clinical visits and by email questionnaire 8 years after gastrectomy.	The concentration of 25(OH)D tended to be higher in the pouch group (47 nmol/L compared to 34 nmol/L).
16.	1999	Schmiedl A. et al. [29]	11 males	Bone mineral density and the associated extracellular status of mineral and acid-base metabolism were evaluated in patients 3–18 years after total gastrectomy.	The serum vitamin D level was normal. The level of total ALP was elevated; fasting urine pH and calcium were low, while phosphorus and net acid were high. The authors concluded that over the long-term gastrectomy evokes low BMD, but not hyperparathyroidism and deranged vitamin D metabolites.
17.	1995	Wu Y.W. et al. [30]	1 patient	Single patient with osteomalacia secondary to vitamin D deficiency after gastrectomy for gastric cancer.	After vitamin D treatment, the appearance of repeated bone scintigram was normalized.
18.	1994	Kobayashi S. et al. [31]	29 men who had undergone Billroth I gastrectomy and 19 men who had undergone Billroth II gastrectomy	The patients were examined for their calcium-regulating hormones and bone mineral content after surgery.	The 24,25(OH)_2_D concentration was reduced and the 25(OH)D and 1,25(OH)_2_D concentrations were increased in the Billroth II group. The authors suggested that post-gastrectomy bone metabolic disease is not due to vitamin D deficiency, but may be due to reduced calcium absorption in the intestine. Serum calcium and phosphate concentrations in patients with Billroth I and Billroth II were normal. The Billroth II group had an elevated serum alkaline phosphatase level and a reduced bone mineral content.

BMD—bone mineral density; 1,25(OH)_2_D—1,25-dihydroxy vitamin D; 25(OH)D—25-hydroxy vitamin D; E2—estradiol; DEXA—dual-energy X-ray absorptiometry; ALP—alkaline phosphatase; BMI—body mass index; RR—relative risk; PERT—pancreatic enzyme replacement therapy; MD/MS—modified microdensitometry method.

## Data Availability

No new data were created or analyzed in this study. Data sharing is not applicable to this article.

## Abbreviations

1α(OH)D	1αhydroxyvitamin D
1α,25(OH)_2_D	1α,25-dihydroxyvitamin D, calcitriol
24,25(OH)_2_D	24,25-dihydroxyvitamin D
25(OH)D	25-hyroxyvitamin D, calcidiol
ALP	alkaline phosphatase
BMD	bone mineral density
BMI	Body Mass Index
D2-LND	D2 lymphadenectomy
DEXA	dual-energy X-ray absorptiometry
E2	estradiol
FAP	familial adenomatous polyposis
GAPPS	gastric adenocarcinoma and proximal polyposis of the stomach
HDGC	hereditary diffuse gastric cancer
IL	interleukin
MD/MS	modified microdensitometry method
miRNAs	microRNAs
PERT	pancreatic enzyme replacement therapy
RR	relative risk
UVB	ultraviolet B
VDBP	vitamin D binding protein
